# Correlations between well-being of nurses and psychosocial working conditions – a descriptive cross-sectional study

**DOI:** 10.3389/fpubh.2024.1443015

**Published:** 2024-07-24

**Authors:** Katarzyna Tomaszewska, Krystyna Kowalczuk, Bożena Majchrowicz

**Affiliations:** ^1^Department of Nursing, Institute of Health Protection, The Bronisław Markiewicz Academy of Applied Sciences, Jarosław, Poland; ^2^Department of Integrated Medical Care, Medical University of Bialystok, Białystok, Poland; ^3^Department of Nursing, Institute of Health Protection, State Academy of Applied Sciences, Przemyśl, Poland

**Keywords:** nurse, working conditions, physical strain, mental strain, nurses

## Abstract

**Introduction:**

Work in health care is classified as a difficult profession and nurses are considered among the professional group that is exposed to the permanent impact of occupational stress. Psychosocial working conditions and related hazards are defined as those aspects that have the potential to cause harm to an employee’s mental or physical health. Lack of psycho-physical health well-being reduces job satisfaction and thus job commitment.

**Aim:**

The aim of this study was to assess the overall well-being of nurses and examine the correlation between nurses’ well-being and their assessment of psychosocial working conditions in conjunction with occupational and demographic factors.

**Materials and methods:**

A descriptive cross-sectional survey was conducted among 526 nurses employed in a selected public clinical hospital in Poland. All nurses provided labor during the survey. A diagnostic survey method using the standardized Psychosocial Working Conditions questionnaire based on the demands-control-support stress model was used for measurement.

**Results:**

The examined nurses rated highly job demands (mean 3.46) as well as the scale of desired changes (mean 3.44). The ability to control their work (mean 3.19) and the level of social support (mean 3.21) were rated at a slightly lower level. The scale of well-being was rated highest by respondents (mean 3.68). Several statistically significant correlations (*p* < 0.05) can be observed between the well-being scale and the other scales of psychosocial working conditions across age categories. The least correlated are the well-being and demands scales, although as age increases with higher levels of well-being, the demands scale scores decrease.

**Conclusion:**

The well-being of the examined nurses was closely related to sociodemographic data and the individual scales of the Psychosocial Working Conditions questionnaire. Chronic diseases are associated with greater demands at work and reduced well-being. Respondents who receive higher levels of support at work experience higher levels of well-being.

## 1 Introduction

Mental well-being is considered an important indicator of health in research and policy debates because it reflects a person’s overall assessment of quality of life, happiness and satisfaction. It is an important determinant of individual productivity at both enterprise and societal level (1). Physical well-being is a basic indicator of quality of life, which characterizes the health condition of an individual or social group. It is treated as the well-being of a person who knows one’s potential and understands one’s emotions. At the same time, one is resilient to stress, takes care of one’s body, and has a sense of purpose, connectedness and belonging to the community (2). Lack of well-being in terms of not only mental health, but also physical health lowers the level of job satisfaction and thus commitment to work (1).

Work in health care is classified as a difficult profession due to its complexity, as well as multitasking. Carrying out the work of a nurse due to the specifics of the profession – constant contact with another (sick, suffering, sometimes dying) person causes nurses to be counted among the professional group that is exposed to the permanent impact of occupational stress (3, 4). An undeniable source of stress among medical personnel is the specific organization of work, which is characterized by irregularity, shift work, prolonged periods of work in constant tension or shortage of staff. All these factors consequently translate into physical and mental fatigue (5). The professional work of nurses is inextricably linked to unpredictable situations, during which often the lives of patients are decided by mere seconds. Time pressure contributes to progressive fatigue, reduced work efficiency, as well as a decline in nurses’ psychological well-being (6).

Psychosocial hazards can be defined as those aspects of the design and management of the work process, along with their socio-organizational context, that can cause psychological or physical harm (7, 8). The International Labor Organization defines psychosocial risk factors as the interaction between the content of work, the management and organization of the work process and other organizational and environmental conditions on the one hand, and the needs and competencies of workers on the other (9). Psychosocial risks can be defined more simply as those aspects of the work process with their organizational and social context that have the potential to cause psychological or physical harm and are associated with the experience of occupational stress (10, 11). Psychosocial working conditions are a key determinant of work stress, an important mediator in the path between shift work and health (12). Therefore, the work environment is very important for employee health and productivity (13). Thanks to decades of extensive research on occupational health and safety, the physical and psychosocial work conditions that pose risks and resources are now well understood (14). Psychosocial occupational hazards are not always immediately apparent and are sometimes difficult to diagnose, but like physical hazards they are controllable (e.g., through psychosocial risk management) (15).

Important factors associated with stress at work are its demands, job tension and uncertainty, and resources such as job control and social support. High job demands, low levels of job control and poor social support are associated with various outcomes of poor health, such as poor mental health and sick leave (16). In contrast, high demands combined with high levels of control lead to better well-being, learning, motivation and skill development. Various models of stress at work commonly maintain that high demands at work do not necessarily negatively affect psychological well-being when combined with sufficient resources and rewards (2). According to Siegrist’s effort-reward imbalance model, high levels of effort expended combined with low reward can lead to strong negative emotions and stress reactions. Job insecurity and employment anxiety are considered determinants of poor psychological well-being, although the mechanisms of how these problems affect employee well-being are unclear. Nevertheless, perceived job insecurity has been linked to reduced psychological well-being. The risk of losing one’s job can be just as stressful as actually losing one’s job, as the uncertainty of the situation makes it difficult to manage the situation and respond appropriately (17).

Nursing requires commitment to the work and concern for the welfare of the patient (5, 18). Two key determinants of the quality of nurses’ psychosocial work environment are workload and the quality of relationships with other health care professionals. Regarding workload, studies have shown that even when nurses manage to effectively prioritize and adjust their work practices under high time pressure, the increased risk of adverse consequences for patients remains highly unsatisfactory. In terms of social relationships in the workplace, some of the key characteristics include the ability to work with competent colleagues, supportive relationships with management, and working in cultures that promote continuous improvement and skill development (19).

The presented study can expand the knowledge of the factors on which the well-being of the studied group of nurses depends. It can also show that the provision of proper psychosocial working conditions is a very important element in the job satisfaction of the examined nurses.

The aim of this paper was to assess the overall well-being of nurses and to investigate the correlation between nurses’ well-being and their assessment of psychosocial working conditions in relation to occupational and demographic factors. Based on the purpose of the study, the following research problems were formulated:

1. Is the assessment of well-being correlated with selected sociodemographic factors?
2. Are there correlations between well-being and individual scales of the Psychosocial Working Conditions questionnaire?
3. Are there correlations between the prevalence of chronic diseases among respondents, well-being and the individual scales of the Psychosocial Working Conditions questionnaire?

Analysis of the obtained results would allow us to understand what the respondents’ well-being depends on, whether the examined nursing staff expects changes in their work environment, and if so, what aspects they concern. At the same time, thanks to the knowledge of what factors affect their well-being, it will be possible to outline strategies for improvement at individual workplaces.

## 2 Materials and methods

### 2.1 Research design

In the present study, a survey was conducted among nurses employed at a selected clinical hospital in Poland’s public health sector. The survey was conducted in May 2023. This survey-based, descriptive cross-sectional study was carried out to assess respondents’ knowledge of psychosocial working conditions.

### 2.2 Research tools

Authors used the standardized Psychosocial Working Conditions questionnaire to measure psychosocial working conditions, which is based on the demands-control-support stress model. According to this model, stress at work is the resultant of three main characteristics of work: the magnitude of job demands; the possibility of control, i.e., the ability to influence the work and its conditions; and social support, i.e., the feeling of being able to receive help in everyday and difficult situations. The most unfavorable situation from the point of view of stress is one in which high demands are accompanied by low levels of control and social support.

The questionnaire consists of five theoretical scales:

1. Demands scale (W) – what demands does your job make?
2. Control scale (K) – to what extent can you influence what happens at work?
3. Social support scale (WS) – what support and help can you count on?
4. Well-being scale (D) – what is your well-being?
5. Scale of desired changes (PZ) – do you expect any changes at work?

In addition to the theoretical scales, empirical scales were developed. They consist of three scales on requirements:

1. intellectual requirements,
2. psycho-physical requirements and those arising from safety responsibilities,
3. requirements resulting from role conflict and overload.

Within the scales measuring control, there are two empirical scales:

1. behavioral control,
2. cognitive control.

The scale to assess social support includes two empirical scales:

1. support from superiors,
2. support from co-workers.

The scale for well-being distinguishes two empirical scales:

1. physical well-being,
2. psychological well-being.

The advantage of the Psychosocial Working Conditions questionnaire is the norms obtained from 8 different occupational groups, which allow the results to be compared with other occupations. A total of 3,992 people were examined from the following occupational groups: banking and insurance specialists, nurses, construction workers, salespeople, government officials, IT specialists, public transportation drivers and teachers. The norms of the questionnaire make it possible to relate the results of the examined group to all of the aforementioned occupational groups combined. The internal consistency indices (Cronbach’s alpha) of the individual theoretical scales are high. They range from 0.74 to 0.87 for the requirements scale, 0.79–0.86 for the control scale, 0.92–0.96 for the social support evaluation scale, 0.88–0.91 for the well-being scale, and 0.88–0.93 for the need for change scale in each professional group. For the purposes of this study, scores were calculated for each scale and subscale according to the coding rule and key provided by the authors. The scores for the answers to the questions included in the scale were then summed and the obtained values were compared with the norms (20).

### 2.3 Participants

In the present study, a survey was conducted among 526 nurses employed at a selected clinical hospital in Poland’s public health sector. The survey was conducted by a diagnostic survey method using Psychosocial Working Conditions Questionnaire. Respondents had varying levels of seniority and education. Each respondent independently and voluntarily completed the survey questionnaire and gave written consent to participate in the study, and each respondent received information about the processing of respondents’ personal data. The consents and survey questionnaires are in the possession of the author of the paper. Initially 752 questionnaires were distributed, 526 were accepted and correctly completed, which accounted for 69.95%. The criteria for inclusion in the study were current employment and consent to participate in the study. The questionnaires were left in the nursing rooms and after completion were collected by the authors of the study.

### 2.4 Statistical analysis

In the analysis of the collected material, descriptive statistics were used to present the most important information about the variables analyzed in the study and the group of respondents. The choice of statistical methods was determined by the nature of the characteristics under consideration. In the case of the study of the impact of a trait of a nominal nature on the evaluation of working conditions, statistical analysis was reduced to a comparison of average values in the separated groups. Correlations between ordinal or quantitative variables (during the unfulfilled conditions of using parametric tests) were made using Spearman’s rho coefficient, which indicates the intensity of the relationship and its direction – positive or negative. The resulting value ranges from −1 to 1, with (−1) indicating a perfect negative correlation and (1) a perfect positive correlation. Having previously met the assumptions provided for parametric tests, the analyses used r-Pearson correlations and stepwise linear regression. The r-Pearson correlation coefficient can take values from −1 to 1. Based on the numerical value, we can infer the strength of the relationship – the closer the value is to zero, the weaker the strength of the relationship. The analysis was performed using the IBM SPSS 29.0 package with the Exact Tests module. All relationships/correlations/differences are statistically significant when *p* ≤ 0.05.

### 2.5 Ethical procedures

The participation of nurses in the study was voluntary and anonymous. The study was conducted in accordance with the ethical standards set forth in the Declaration of Helsinki (64th WmA General Assembly, Fortaleza, Brazil, October 2013) and in accordance with Polish legal regulations. The application was favorably approved by the Bioethics Committee of the State Academy of Applied Sciences in Przemysl (KBPANS No. 06/2023).

## 3 Results

The characteristics of the study group are shown in Table 1.

**Table 1:** Characteristics of the study group.

Variable		Respondents (*N* = 526)	
		Frequency (*N*)	Percentage (%)
Gender	Female	507	96.4
	Male	19	3.6
Age (years)	22–30	98	18.6
	31–40	133	25.3
	41–50	189	35.9
	>50	106	20.2
Education	University degree	329	62.5
	Medical vocational school	118	22.4
	Medical high school	79	15.0
Job seniority (years)	1–5	128	24.3
	6–10	78	14.8
	11–20	119	22.6
	21–30	147	28.0
	>30	54	10.3
Job seniority in the currently held position (years)	1–5	133	29.1
	6–10	93	17.7
	11–20	125	23.8
	21–30	114	21.7
	>30	41	7.8
BMI	Underweight	8	1.5
	Normal weight	292	55.5
	Overweight	173	32.9
	Obesity	53	10.1
Do you work in a managerial position?	No	463	88.0
	Yes	63	12.0
Do you suffer from any chronic illnesses?	No	396	75.3
	Yes	130	24.7
How many days of work have you missed in the last year due to sickness?	0	161	30.6
	1–10	144	27.4
	11–20	186	35.4
	21–30	22	4.2
	>30	13	2.5

The mean age of the nurses examined was 41.82. The mean BMI score indicates that the respondents are of normal weight. During the past year, the respondents’ absenteeism from work due to sickness averaged 8.4 days.

In the results obtained, the higher the mean (range from 1 to 5), the higher the level of requirements (W), the higher the range of control (K), the higher the level of perceived social support (WS), the higher the level of well-being (D) and the higher the level of desired changes at work (PZ).

The nurses examined rated highly the demands made by the job (mean 3.46) as well as the scale of desired changes (mean 3.44). The ability to control their work (mean 3.19) and the level of social support (mean 3.21) were rated at a slightly lower level. The scale of well-being was rated highest by respondents, with a mean of 3.68. Detailed results are presented in Table 2.

**Table 2:** Mean scores of individual scales of the psychosocial working conditions questionnaire.

	Mean	Median	Standard deviation	Minimum	Maximum
W – Demands scale (1–5)	3.46	3.44	0.39	2.36	4.68
K – Control scale (1–5)	3.19	3.20	0.45	1.80	4.65
WS – Social support scale (1–5)	3.21	3.25	0.75	1.00	5.00
D – Well-being scale (1–5)	3.65	3.68	0.53	1.86	4.86
PZ – Scale of desired changes (1–5)	3.44	3.50	0.70	1.00	4.95

Several statistically significant correlations (*p* < 0.05) can be observed between the well-being scale and the other scales of psychosocial working conditions across age categories. The least correlated are the well-being and demands scales, although as age increases with higher levels of well-being, the demands scale scores decrease. In addition, the clear values of the correlation coefficients inform that the higher the well-being scores, the higher the scores of control and social support, and lower scores of the scale of desired changes are observed. Between the well-being scale and the scales of control and desired changes, a trend can be seen that there is greater strength of the relationship in older age groups (Table 3).

**Table 3:** Correlations between respondents’ age, well-being and individual scales of the psychosocial working conditions questionnaire.

Scale		D – Well-being scale (1–5)			
Age (years)		22–30	31–40	41–50	Above 50
W – Demands scale (1–5)	Pearson’s correlation	−0.153	−0.167	−0.165^*^	−0.234^*^
	Frequency (*N*)	98	133	183	106
K – Control scale (1–5)	Pearson’s correlation	0.391^**^	0.354^**^	0.455^**^	0.97^**^
	Frequency (*N*)	98	133	183	106
WS – Social support scale (1–5)	Pearson’s correlation	0.433^**^	0.244^**^	0.416^**^	0.328^**^
	Frequency (*N*)	98	133	183	106
PZ – Scale of desired changes (1–5)	Pearson’s correlation	−0.311^**^	−0.329^**^	−0.328^**^	−0.406^**^
	Frequency (*N*)	98	133	183	106

^**^Correlation significant at the 0.01 level (two-tailed).

^*^Correlation significant at the 0.05 level (two-tailed).

Considering the two types of educational degree, similar values of correlation coefficients are observed between well-being and the scales of demands, control, social support and desired changes. The most pronounced relationship strengths that are statistically significant (*p* < 0.05) are between the well-being scale and the scales of control, social support and the scale of desired changes (Table 4).

**Table 4:** Correlations between respondents’ education, well-being and individual scales of the psychosocial working conditions questionnaire.

Scale		D – Well-being scale (1–5)	
Education		Secondary	Higher
W – Demands scale (1–5)	Pearson’s correlation	−0.180^*^	−0.210^**^
	Frequency (*N*)	204	320
K – Control scale (1–5)	Pearson’s correlation	0.394^**^	0.371^**^
	Frequency (*N*)	204	320
WS – Social support scale (1–5)	Pearson’s correlation	0.320^**^	0.358^**^
	Frequency (*N*)	204	320
PZ – Scale of desired changes (1–5)	Pearson’s correlation	−0.309^**^	−0.362^**^
	Frequency (*N*)	204	320

^**^Correlation significant at the 0.01 level (two-tailed).

^*^Correlation significant at the 0.05 level (two-tailed).

Analyzing the correlations between well-being and the other scales relating to psychosocial working conditions in each category of job seniority, the strongest value of the correlation coefficient, which is statistically significant (*p* < 0.05), informs that in the group of those who have been working for 6–10 years, the higher the well-being scores, the higher the control scale scores. Similar correlations apply to the other seniority groups, but they are already less pronounced. In addition, higher levels of well-being are associated with higher levels of social support and lower levels of desired changes, but less clear statistically significant correlations in this regard can be seen in the group of those working from 11 to 20 years (Table 5).

**Table 5:** Correlations between respondents’ job seniority, well-being and individual scales of the psychosocial working conditions questionnaire.

Scale		D – Well-being scale (1–5)				
Length of service in current position (years)		1–5	6–10	11–20	21–30	Above 30
W – Demands scale (1–5)	Pearson’s correlation	−0.229^**^	−0.101	−0.010	−0.391^**^	−0.119
	Frequency (*N*)	145	93	125	114	41
K – Control scale (1–5)	Pearson’s correlation	0.333^**^	0.548^**^	0.348^**^	0.443^**^	0.319^*^
	Frequency (*N*)	145	93	125	114	41
WS – Social support scale (1–5)	Pearson’s correlation	0.433^**^	0.362^**^	0.267^**^	0.343^**^	0.398^**^
	Frequency (*N*)	145	93	125	114	41
PZ – Scale of desired changes (1–5)	Pearson’s correlation	−0.334^**^	−0.435^**^	−0.207^*^	−0.425^**^	−0.327^*^
	Frequency (*N*)	145	93	125	114	41

^**^Correlation significant at the 0.01 level (two-tailed).

^*^Correlation significant at the 0.05 level (two-tailed).

There is a higher strength of the relationship between well-being and the scales of control and social support among those who work in managerial positions compared to those in lower positions. Higher values of one variable are associated with higher values of the other variable. For both managerial and non-managerial positions, a clear and negative statistically significant correlation (*p* < 0.05) is found between the well-being scale and the scale of desired changes (Table 6).

**Table 6:** Correlations between occupation, well-being and individual scales of the psychosocial working conditions questionnaire.

Scale		D – Well-being scale (1–5)	
Do you work in a managerial position?		No	Yes
W – Demands scale (1–5)	Pearson’s correlation	−0.166^**^	−0.223
	Frequency (*N*)	461	63
K – Control scale (1–5)	Pearson’s correlation	0.406^**^	0.518^**^
	Frequency (*N*)	461	63
WS – Social support scale (1–5)	Pearson’s correlation	0.342^**^	0.534^**^
	Frequency (*N*)	461	63
PZ – Scale of desired changes (1–5)	Pearson’s correlation	−0.344^**^	−0.276^*^
	Frequency (*N*)	461	63

^**^Correlation significant at the 0.01 level (two-tailed).

^*^Correlation significant at the 0.05 level (two-tailed).

Among those who have chronic diseases compared to those who do not have such diseases, there is a more pronounced strength of the relationship statistically significant (*p* < 0.05) between the scale of well-being and the sum of desired changes. Higher levels of well-being are associated with lower levels of desired changes. In addition, quite clear and similar values of correlation coefficients in the two analyzed groups relate to the scale of well-being and the scale of control and social support. The higher the level of well-being, the higher the scores of the scale of control and social support (Table 7).

**Table 7:** Correlations between respondents’ prevalence of chronic diseases, well-being and individual scales of the psychosocial working conditions questionnaire.

Scale		D – Well-being scale (1–5)	
Do you suffer from chronic diseases?		No	Yes
W – Demands scale (1–5)	Pearson’s correlation	−0.135^**^	−0.246^**^
	Frequency (*N*)	396	130
K – Control scale (1–5)	Pearson’s correlation	0.390^**^	0.427^**^
	Frequency (*N*)	396	130
WS – Social support scale (1–5)	Pearson’s correlation	0.353^**^	0.375^**^
	Frequency (*N*)	396	130
PZ – Scale of desired changes (1–5)	Pearson’s correlation	−0.281^**^	−0.443^**^
	Frequency (*N*)	396	130

^**^Correlation significant at the 0.01 level (two-tailed).

## 4 Discussion

The aim of the study was to assess the overall well-being of nurses and examine the correlation between nurses’ well-being and their assessment of psychosocial working conditions in conjunction with occupational and demographic factors.

An analysis of the relationship between ratings of various aspects of nurses’ psychosocial working conditions was conducted. The respondents rated the scale of well-being highest and at a comparable high level rated the demands of their work as well as the scale of desired changes. The ability to control their work and the level of social support were rated at a slightly lower level. There are a few statistically significant correlations between the length of job tenure and the scores of the various scales, but each is characterized by negligible strength of association. It should also be noted that there are no statistically significant correlations between age and psychosocial working conditions. Older employees with a high sense of well-being showed less need for change than their younger counterparts. Almeida et al. proved that nurses’ levels of job well-being differed significantly in terms of education level, job satisfaction and life satisfaction (6). This was corroborated by Carneiro and Bastos, proving that factors related to well-being at work include sociodemographic data and interactions within the treatment team, interaction between personal and professional life, and organizational commitment (21). Self-study among people with chronic diseases showed a stronger correlation between well-being and the scale of desired changes. Other studies have found that middle-aged individuals were characterized by poorer health and greater exposure to psychosocial risks (12, 22, 23). Bujacz et al. demonstrated that mid-career nurses in work environments characterized by low autonomy and support tended to report poorer health outcomes (19), while other authors have found that nurses working in hospitals find that their jobs place high demands on them, while their sense of control over their work is reduced, which predisposes them to very high levels of stress (7) and poor quality of life (24). A study in Norway using data from the WorkSafeMed survey, statistically found only differences between the time control scale and social relationships (3).

Our own study found that lower levels of education were associated with lower scores on the control scale, the social support scale and the well-being scale, but the strengths of the association between the variables were insignificant. A study by Misiak et al. found that better-educated respondents rated the level of demands at work as higher, but they were also more satisfied in the well-being category (5). Lorber et al. proved that the level of well-being differed significantly according to the level of education, and seniority and place of work (10), which is confirmed by other studies (25).

Gustafsson et al. showed that by examining three dimensions of psychosocial working conditions: job demands, job control and job support, the problems of presenteeism and low work capacity among many health and care workers can be alleviated by reducing psychosocial demands (11).

In our own research, the mean period of respondents’ incapacity to work on sick leave was more than 8 days. A survey of nurses in Sweden found that the high rate of sickness absence among care workers in Sweden can be reduced if simultaneous exposure to high psychosocial and physical challenges is avoided. Management policies to reduce time pressure, improved lifting aids and measures to prevent uncomfortable working positions are recommended (26). In another study, due to the numerous stressors and psychosocial working conditions, it was claimed that employers should make an effort to modify them (27) which is supported by other studies (28). A study by Ersin et al. found a positive, weakly significant relationship between nurses’ psychological well-being and their perception of social support (29) and increasing social support from fellow nurses is an effective way to deal with the negative effects of nurses’ emotional demands. Younger nurses tended to have higher emotional demands and lower social support from colleagues (30). Social support is a key resource in the context of nursing work, with beneficial effects on well-being (e.g., reducing role stress) and job satisfaction (31). Factors of the work environment, such as adequate staffing, good cooperation with physicians, support from management, and professional autonomy, significantly affect nurses’ assessment of patient safety (32). Given the large number of published studies on the impact of psychosocial working conditions on workers’ health, it is important to consider that for outcomes such as cardiovascular disease and depression, their results show that respondents reporting excessive workloads, imbalance between commitment and reward, job insecurity and long working hours are at increased risk of psychosocial strain (33).

## 5 Limitations of the study

A strength of the study is the number of examined nurses participating in the survey. Limitations characteristic of cross-sectional studies apply. The survey was conducted in a single clinical hospital at a specific time. In addition, during the survey, there was a possibility of an exchange of opinions among the nurses, which may have influenced their responses.

## 6 Conclusion

The study has proved that the higher the well-being scores, the higher the control and social support scores, and observed lower scores on the desired change scale. Older employees with high levels of feelings of well-being showed less need for change than their younger counterparts. Education, length of service and position held did not significantly differentiate the correlation between well-being and the other scales of psychosocial working conditions. Among those with chronic diseases, there was a stronger correlation between well-being and the scale of desired changes. Nurse managers and executives should consider the above findings when developing strategies to improve the work environment, aiming to reduce workload and retain nurses in the profession. It is also worth conducting multi-center studies to generalize the findings and gain better insight into the psychosocial aspects of nurses’ work, which are the basis of job satisfaction which is inextricably linked to higher quality of services provided.

## Data availability statement

The raw data supporting the conclusions of this article will be made available by the authors, without undue reservation.

## Ethics statement

The studies involving humans were approved by the Bioethics Committee of the State Academy of Applied Sciences in Przemysl. The studies were conducted in accordance with the local legislation and institutional requirements. The participants provided their written informed consent to participate in this study.

## Author contributions

KT: Conceptualization, Data curation, Formal analysis, Investigation, Methodology, Project administration, Resources, Software, Supervision, Validation, Visualization, Writing – original draft, Writing – review & editing. KK: Conceptualization, Data curation, Formal analysis, Investigation, Methodology, Project administration, Resources, Software, Supervision, Validation, Visualization, Writing – original draft, Writing – review & editing. BM: Conceptualization, Data curation, Formal analysis, Investigation, Methodology, Resources, Supervision, Validation, Writing – original draft, Writing – review & editing.
